# Ethics in occupational health: deliberations of an international workgroup addressing challenges in an African context

**DOI:** 10.1186/1472-6939-15-48

**Published:** 2014-06-23

**Authors:** Leslie London, Godfrey Tangwa, Reginald Matchaba-Hove, Nhlanhla Mkhize, Remi Nwabueze, Aceme Nyika, Peter Westerholm

**Affiliations:** 1 Centre for Occupational and Environmental Health Research, University of Cape Town, Cape Town, South Africa; 2 University of Yaounde and Cameroon Bioethics Initiative (CAMBIN), Yaounde, Cameroon; 3 School of Public Health, University of Botswana, Gaborone, Botswana; 4 University of KwaZulu-Natal, Durban, South Africa; 5 University of Southampton, Southampton, United Kingdom; 6 Public Health Projects in Africa, Harare, Zimbabwe; 7 Faculty of Health Sciences, University of the Witwatersrand, Johannesburg, South Africa; 8 University of Uppsala, Uppsala, Sweden

**Keywords:** Ethics, Occupational health, Occupational health practitioner, African philosophy, Globalisation, Stigma, Consent, Culture, Autonomy, Ubuntu, Harmony, Identity

## Abstract

**Background:**

International codes of ethics play an important role in guiding professional practice in developing countries. In the occupational health setting, codes developed by international agencies have substantial import on protecting working populations from harm. This is particularly so under globalisation which has transformed processes of production in fundamental ways across the globe. As part of the process of revising the Ethical Code of the International Commission on Occupational Health, an Africa Working Group addressed key challenges for the relevance and cogency of an ethical code in occupational health for an African context through an iterative consultative process.

**Discussion:**

Firstly, even in the absence of strong legal systems of enforcement, and notwithstanding the value of legal institutionalisation of ethical codes, guidelines alone may offer advantageous routes to enhancing ethical practice in occupational health. Secondly, globalisation has particularly impacted on health and safety at workplaces in Africa, challenging occupational health professionals to be sensitive to, and actively redress imbalance of power. Thirdly, the different ways in which vulnerability is exemplified in the workplace in Africa often places the occupational health professional in invidious positions of Dual Loyalty. Fourth, the particular cultural emphasis in traditional African societies on collective responsibilities within the community impacts directly on how consent should be sought in occupational health practice, and how stigma should be dealt with, balancing individual autonomy with ideas of personhood that are more collective as in the African philosophy of *ubuntu*. To address stigma, practitioners need to be additionally sensitive to how power imbalances at the workplace intersect with traditional cultural norms related to solidarity. Lastly, particularly in the African context, the inseparability of workplace and community means that efforts to address workplace hazards demand that actions for occupational health extend beyond just the workplace.

**Summary:**

A stronger articulation of occupational health practice with advocacy for prevention should be an ethical norm. Ethical codes should ideally harmonize and balance individual and community needs so as to provide stronger moral authority guidelines. There is a need to consider an African Charter on Bioethics as complementary and strengthening of existing codes for the region.

## Background

There has been increasing attention to questions of professional ethics in the field of occupational health over the past two decades. This surge in interest has been partly driven by the broader engagement of the health professions with key moral challenges facing practitioners in the field [1-3] and from growing attention to matters of research ethics. The latter development, first prompted by the revelation of Nazi medical atrocities in the Second World War, and by research scandals linked to Tuskagee, Willowbrook and Guatemala, has seen a recognition that more careful elaboration of ethical codes is required to address the realm of international health research in the 21^st^ century [4-6]. However, in the occupational health field, this interest has also been driven by the changing nature of work in both developed and developing countries, largely linked to globalisation of production and the challenges associated with health and safety under globalisation [7-10]. Many of these challenges raise complex ethical questions demanding careful reflection so as to inform best practice. By and large, professions develop ethical guidelines in order to support practitioners to negotiate these difficult questions, seeking to update such guidelines periodically to ensure consonance with the latest developments in ethical discourse.

For developing countries, ethical guidelines issued by international bodies frequently serve as the only source of ethical standards in settings with weak regulatory infrastructure and/or limited ethical expertise. This mimics a broader reliance on guidance documents and norms addressing health emanating from intergovernmental bodies such as the World Health Organisation (WHO), International Labour Organisation (ILO) and other United Nations (UN) family organizations. The integrity, relevance and contemporanousness of guidelines from international agencies are therefore critical for protecting the health and well-being of patients, communities and users of health care globally.

In line with this understanding, the International Commission on Occupational Health (ICOH), an international non-governmental professional society that aims to foster the scientific progress, knowledge and development of occupational health and safety in all its aspects, resolved to update its 2002 Code of Ethics [11] through a globally consultative process in 2009. The process intended, inter alia, to increase the relevance of the code to working populations from the most vulnerable settings across the globe, and established networking relationships with occupational health professionals in Latin America, Asia and Africa to solicit locally relevant input to a revision of its global ethical code.

In Africa, a workgroup, drawing on participants from 5 countries (South Africa, Cameroon, Nigeria, Zimbabwe and Botswana) and a range of disciplinary fields (occupational health, bioethics, psychology, law and philosophy), was established in 2010 to address ethical issues contextualised by experience of occupational health practice on the African continent. Iterative discussions were held over a period of three years from 2010 to 2013, including two conference symposia to present and get feedback on developing ideas. This paper reports the consensus of the workgroup regarding key ethical challenges for occupational health practice in the African context.

First, we outline the relevance of this matter for countries in Africa, reflecting on the place of ethical codes in the absence of regulations and locate the discussion in the context of both the relevance of globalisation to occupational health ethics in Africa and the broader governance challenges in Africa related to lack of political democracy and public accountability. Then, we examine four particularly challenging dilemmas in occupational health practice in Africa: workplace vulnerability; consent; stigma; and the frequent absence of a distinction between work and home. From this, we conclude with a suggestion that, in addressing the challenges for occupational health ethics particular to the African context, the concept of harmony or relational solidarity, embedded in traditional views of personhood being socially based may be helpful. This concept, variously expressed across the traditional cultures of Africa, in effect establishes a dialectic relationship and balance between individual autonomy and communal values. One of its best and most articulate expressions is found in the Southern Africa philosophy of *Ubuntu*[12-15] more fully expressed in the adage Umuntu, Ngumuntu Ngabantu (a person is a person because of other persons). Among the Nso' of the Northwestern region of Cameroon, the same concept is expressed verbatim in the Lamnso' proverb “Wir dze wir bih wir” while the Igbo of eastern Nigeria express it more metaphorically - If a person feels an itch in the back, he calls his fellow to scratch him; an animal scratches itself against a tree. Given particular visibility in the realm of public engagements over social justice and transformation in South Africa by Archbishop Desmond Tutu, *ubuntu* has been framed as the essence of being human, because it recognises that one’s humanity is inextricably bound with others’ humanity [15]. It has come to be seen as a particularly African contribution to considerations of public values globally [16] and, for this paper, serves as a particularly useful framework to analyse some of the key ethical challenges in occupational health practice in the region.

## Discussion

### Ethical Codes in Occupational Health Practice – the relevance for Africa

Whereas the research arena has relatively well-accepted ethical rules now regarded as standard (for example, informed voluntary consent is essential for participation in medical research; the ability and capacity to exercise voluntary free choice must be ensured; research must have benefits or prospect of benefits for the participants and their wider community; research protocols must be approved by an independent ethics review committee; the burden for demonstrating ethical research rests squarely with the researcher), there is less attention to the ethical implications of practice, and particularly so in the occupational health field. Even where ethical rules are in place, they require legal backing and underpinnings [17,18] for the following reasons: human beings everywhere suffer from important limitations with regard even to what they themselves freely accept as right and reasonable to do; there is the problem of akrasia/weakness of will or backsliding in moral matters; there are the problems of self-interest and egoism, abuse and misuse of power/expertise and the enticement of economic advantage; social regulation and particularly legislation is a powerful aid to morally correct behavior; the law is one of the ‘road companions’ of morality, the others being religion and social customs.

In actual fact, only fear of legal sanctions prevents some people from moral wrong-doing, especially in advanced complex modern societies. Modern humans tend to act with impunity when they know they can escape sanctions. This is one reason that many researchers from the advanced countries strictly respect and comply with ethics regulations at home but violate them easily abroad. The research scandals that have recently been recorded in the developing world, particularly in trials in sub-Saharan Africa, involving Trovan and Tenofovir, are sufficiently illustrative of this fact. Such scandals today hardly occur in the developed world, not because the temptations to do unethical research are absent, but most likely because of fear of legal consequences.

In any case, it is a fact that the majority of African countries do not have legally binding and enforceable frameworks for medical practice or research on humans and some of those which do exist are weak or lack enforcement capacity. Outside of South Africa, with a fairly robust legal framework, only a handful of the other 56 African countries, such as Uganda, Malawi, Nigeria, and Tanzania, have even attempted providing such a framework. Moreover, broader democratic processes in many countries in the region remain fragile, with a limited culture of accountability and respect for the rule of law in ensuring good governance. In many African countries, international ethical guidelines are therefore the first and most important reference document for professional conduct or medical research on humans. In such a situation, it is important to draw on the full advantages of guidelines in the absence of laws.

Following ethical guidelines without legal constraints does have some advantages. First of all, the law may limit the possibility of courses of action based on common sense or rationality, let alone allowing for justifiable exceptions to general rules. Secondly, acting from moral conviction is superior to acting from legal constraint. Thirdly, ethical guidelines are equally and uniformly applicable in all countries and within all contexts, subject only to local adaptation and coloration. Fourthly, the legal situation of African countries is complex and diverse, some having bi-jural and others tri-jural systems, depending on colonial backgrounds and influences. But, no matter how good and effective, a law cannot be quoted, let alone enforced, outside of the territory of its application and jurisdiction. In fact, some African countries, relying only on ethical guidelines – Ghana and Sudan for example – seem able to regulate medical research quite effectively. This situation may perhaps best be captured in the African metaphor that for want of a knife some people peel their roasted potatoes with fingernails and can sometimes do it better and more efficiently than those using a knife!

### Globalisation

One factor evident in the preceding discussion is how local practice is inevitably shaped by global forces. Globalization has been defined as the intensification of economic, political, social and cultural connections across borders [19]. The creation of economic and other relations superseding national boundaries is not new, nor is economic interdependence between countries, but what is novel is the unique intensity and velocity of change. There is no state in the world untouched by globalisation. Globalization as an economic process, involving production, distribution, management and finance related to the production of goods and services is an important force that influences organizations throughout the world competing for trade, creating expectations for high performance, high quality and low cost. During recent decades international movements of goods and services have accelerated exponentially and trade barriers have been systematically removed, particularly in free trade zones. Participants in globalization such as international organizations, states, national institutions, undertakings and individuals are not passive objects under globalisation but most are also active players shaping and influencing patterns of ongoing change processes.

What is striking about globalisation is, firstly, how pervasive it has become – across the globe and across sectors. For undertakings seeking to benefit from globalisation, there are many implications for human resource management, since, to remain competitive, human resource management is under pressure to adopt and support policies promoting labour flexibility or forms of workplace production that place the rapid transfer of technologies and products at the centre of workplace organisation [20]. Secondly, it is well recognised that globalization, particularly in its neoliberal application, is uneven in terms of cross-national strength, geographical scope and national or local depth of change process. As a result, globalization creates insiders and outsiders, integration and fragmentation, and winners and losers. Those who are able to take advantage of globalization, usually those already with power and resources, gain at the expense of those unable to modernise or use opportunities, usually those who are already marginalised, who become even more powerless.

One characteristic of neoliberal globalisation, however, is that global integration is associated with pressures on nation-states to surrender important aspects of their sovereignty, often leading to weakening of the democratic roles of citizens and threats to basic conditions of health, including workplace health and safety.

In the African context, the advent of structural adjustment programmes imposed by international financing organisations in the nineties, with the introduction of free trade zones (also known as export processing zones or special economic zones) increased the spectrum of ‘unregulated’ work, a phenomenon worsened with the recent global economic crisis. This is because many workers are engaged in informal sector production operating outside of formal regulations, and this sector, along with undocumented migrant labour and child labour, part-time work, temporary work and casual work has expanded as formal employment was squeezed under structural adjustment. Agricultural work, which forms the biggest employment sector for countries in Africa, is particularly vulnerable, being seasonal, low-paid and isolated work. Most workers in these activities are not covered by occupational health and safety and labour regulations, nor has economic globalisation consciously addressed the deregulation and outsourcing of work arising from increased competitve pressures and thinning down of the state. That African economies are so vulnerable to the adverse impacts of globalisation is no coincidence. There are historical reasons, arising from colonial and post-colonial processes, in which this cheap ‘unregulated’ labour was attractive for employers seeking quick profits. With time, some regulation was introduced in some sectors, such as factories and mines. However, the large agricultural and informal sectors remain largely unregulated.

Globalisation is a process not limited to the economic sphere but is also profoundly cultural. Increasing interconnectedness and flows of ideas and power shape what peoples in different parts of the globe think about the world, and their place in this world. Unsurprisingly, in the process of globalization and its related mediatisation, the cores and contents of the culture concept have been modified. Indeed, the process of adopting global ethical codes could just as well be regarded as a kind of cultural project that vernacularises what is global [21], and so shrinks distances between experts, thereby transforming practice and norms in occupational health. This is not to assume that culture should be regarded as fixed, nor as always good and an end in itself. The challenge is therefore how to ensure the ICOH Code [11], which calls on health professionals “to protect and promote workers’ health and wellbeing at work throughout their working lives” can be made real under such a scenario where differences in power are so evident.

### The nature of vulnerability in an African context

Vulnerability to workplace hazards is related to the inability of workers to avoid risk or to change situations where they (or their families) are faced with hazardous exposures. The presence of differentials in power at the workplace and its adverse influence on the protection of workers’ health and safety is well recognised [22,23]. For example, where management perceive the protection of workers’ health as a cost to production, the drive for profits may act as an obstacle to the adoption of measures to increase safety and reduce risks at work [24]. Workers who are aware of workplace hazards may be reluctant to act to avoid or limit exposures for fear of victimisation or job loss [25]. Conversely, where trust between management and employees is low, as a result of contestation of power in poorly managed ways, worker uptake of safety measures may be undermined by distrust of management. Indeed, it is not only power differentials between management and workers that are relevant to shaping vulnerability to workplace hazards, but other dimensions of power - race, ethnicity, class, clan and gender – the latter frequently manifested in sexual harassment or sexual violence at the workplace [22,26].

These problems are not unique to the African context. However, three factors are relevant in the African context. Firstly, the levels of vulnerability of working people are far greater than that experienced in developed countries where worker protections are established, if not completely enforced in law. Secondly, the vulnerability experienced by working populations and their families are historically specific to colonial and post-colonial production in the continent. Thirdly, the capacity of states to effect regulation to address or ameliorate vulnerability is greatly restricted under structural adjustment, common in different forms across the continent.

Increased vulnerability to workplace hazards is evident in different ways. For example, large segments of working populations in Africa are migrant and subject to controls that render their ability to act for health and safety limited. Though this is not unique to Africa [27-29], the extent of migrancy is massive and it has played an integral role in regional economies. For example, Southern Africa has seen enormous movement of workers in the extractive industries, living and working under the harshest conditions, resulting in massive burdens of silicosis in former mine workers in the region [30,31]. Evidence presented to South Africa’s Truth and Reconciliation Commission highlighted the way in which black miners were examined collectively for pre-employment medicals in ways that denied their rights to dignity and respect. Moreover, on contracting TB due to the high dust exposure and appallingly poor and overcrowded living conditions, miners were repatriated to their sending areas without any efforts to ensure ongoing treatment of their condition [1]. Not only was this practice an abuse of their rights but it was the source for a massive wave of morbidity and mortality – in driving the TB epidemic across Southern Africa [32] and laying the seeds for the future explosion of the HIV epidemic across the region [33]. Workers, unable to resist this exercise of power, were dehumanised in overcrowded hostels, denied access to care, and adopted a fatalism in their risk perceptions that was integral to the spread of HIV on the mines in South Africa [34]. Similar evidence of how migrancy and mining shared a common pathway for increased risk for HIV has been reported from Tanzania [35] and Ghana [36].

Other forms of vulnerability were particularly race-based in South Africa. For example, early in the AIDS epidemic in South Africa, anecdotal evidence showed that some white employers were able to access the results of HIV tests of their domestic workers in order to dismiss them [37,38]. In the commercial agricultural sector under apartheid, white employers became accustomed to exercising total control over their workers, who were dependent on their employers for housing and access to basic services [39]. Not uncommonly, violence was used as a means to discipline agricultural workers, a situation which generates enormous obstacles for health and safety in commercial agriculture in South Africa [39,40]. Under such circumstances of employer impunity, workers are unlikely to resist unsafe practices, as a result of which, worker health and safety is compromised.

Furthermore, displacement, both internal and external, of large sections of the populations is not uncommon in countries in conflict, a situation that has occurred all too frequently in the continent. Even without overt conflict, other factors such as drought and political crises (e.g. as in Zimbabwe) have led millions of Africans to seek a better, safer and healthier life outside the borders of their own countries. Such workers, operating outside the bounds of legal protection, are particularly vulnerable to exploitation when they get work, since it is usually work in the informal sector, out of sight of any existing regulatory protections. For this reason, informal sector work is often described as vulnerable work [22,29]. Whereas such exploitation may be at the hands of indigenous national populations, it may also be a situation of kin exploiting kin, where immigrants who have established themselves in the receiving country recruit family or clan relations to work under arduous conditions.

Vulnerability to workplace hazards is aggravated by socio-economic factors that induce workers to be more willing to endure risks they would otherwise refuse. High levels of illiteracy or semi-literacy, extreme poverty and high unemployment all aggravate the extent to which workers experience hazardous exposure, and the extent to which, given a particular hazardous exposure, workers are able to protect themselves and their families [23], highlighting the challenges of addressing upstream social determinants of health. Moreover, government capacity to regulate and provide support for health and safety in Africa is severely curtailed by neoliberal economic policies that dominate the region, often manifesting in structural adjustment programmes that render it difficult to build public sector capacity for promoting health and safety [22,41].

In all these above circumstances, the health professional is often caught in the middle of conflicting interests [42-44]. Faced with pressures from employers to put the interests of the company ahead of that of worker-patients, health professionals risk becoming instruments whereby workers’ human rights and their rights to a safe environment are violated [44]. For example, writing about the role of doctors working for the asbestos industry in South Africa, and the “history of neglect by company doctors”, Braun and Kisting point out that “…because company doctors owed their livelihood to mine owners, there was little incentive to diagnose diseases with a longer latency than the worker’s tenure in the mine …” [45]. In another example, doctors providing occupational health services to workers in a mercury reprocessing plant in South Africa failed to take any protective action for almost half the workforce found to exceed the WHO standard for mercury levels in the blood, arguing instead that the WHO standard was “perhaps overly punitive”, and that “exaggerated reports in the press” failed to appreciate that “one can be exposed to mercury and can tolerate it well…”. Here, the providers were not providing undivided allegiance to their patients, but, as a critical colleague observed, were “dancing to the piper’s tune” [46].

Such circumstances demand more nuanced approaches to maintaining ethical practice [47], as recommended in both guidelines for individual practitioners and addressing institutional mechanisms targeting the structural factors at play in a Dual Loyalty (DL) conflict [44]. However, institutional support for occupational health practitioners that might help ameliorate the management of DL conflicts is scarce in Africa [48]. Organisations of occupational health professionals in Africa are small and scattered and have little mandate or capacity to address ethical issues. Training institutions have limited teaching on ethics in Occupational health and there is little culture of recognising the independence of health practitioners in the region.

Additionally, health workers may themselves be targets of violence, particularly given political instability and high levels of crime, so may be themselves victims of the exercise of power, rather than simply caught in the middle.

### Autonomy and consent

One of the challenges faced by occupational health practitioners that has become inevitably more complex with economic globalization, is that relating to informed consent. Even if argued that globalization may have some economic advantages, from a social perspective, it has the potential to worsen the prevailing power differentials within workplaces and between developed and developing countries. Power differentials in the context of poverty, unemployment and lower levels of education or skills not only make workers vulnerable to workplace hazards [23], but also compromise their ability to fully exercise their right to self-determination. The limited ability of workers to give autonomous and informed consent regarding occupational health issues means that the occupational health practitioners may have to try and strike a balance between the need to carry out the assigned task as per contractual agreement with the employer on one hand and to proactively protect the worker’s autonomy on the other, a further illustration of the challenge of dual loyalty [44]. Such a tricky and potentially controversial balancing task necessitates development of appropriate ethical and legal frameworks which should be updated regularly in order to keep abreast with developments in the dynamic employment sector.

Whereas the worker is critical to production and profitability of business ventures, the worker is also usually the weakest stakeholder in the employment environment and one whose autonomy is easily and frequently violated. Truly informed and voluntary consent is therefore a prerequisite for the worker to undergo any occupational health-related tests or procedures, to have results disclosed or to undergo an intervention programme recommended by the occupational health practitioner. These scenarios are generally employer-initiated and are implemented through the occupational health practitioner who reports to the employer. The circumstances under which the consent is to be obtained are different from those surrounding consent for participation in health research, hence the need for guidelines and codes tailor-made for occupational health practice. For health research purposes, the conditions provide better support to autonomous decision-making on the part of the prospective participants since healthcare provision should be separated from health research.

However, particular to most African settings, the process of giving informed consent is rendered more complex, given different cultural conceptions of personhood. In contrast to the “Western” and Anglo-Saxon emphasis in bioethics on the autonomy of the individual, traditional Africa notions of personhood, such as in the concept of ‘*Ubuntu*’ place the individual within a wider set of relationships (‘I am because we are’). This has therefore led African bioethicists to suggest the concept of ‘respect for persons’ as key to consent as opposed to ‘autonomy’ [49]. Extending that to the occupational health context, when dealing with the individual worker, one is in fact dealing with a much broader body of persons who include the immediate family and close community members for whom the decision matters. Such a communitarian culture of African employees, which locates personhood in an interdependent relationship with others in the community, may not be accommodated in the process of obtaining informed consent for occupational health purposes at the workplace where prompt decision-making processes, based on individualistic approaches, are considered to be critical for overall profitability and competitiveness. The right to self-determination implies that if one wants to make any consultations before making a decision one should be given the freedom and time to do so and that workplace medical decision-making should allow workers to consult close relatives before making a decision. However, due to power differentials which prevail at most work places, workers may not be brave enough to ask for time to make such consultations if subjected to hasty decision-making processes.

Given the fact that there is still much work to be done in terms of gender equality and empowerment of women in the world in general and in Africa in particular, the right to self-determination of female workers in patriarchal work-places is prone to deliberate or inadvertent violation. A study conducted in Zimbabwe showed that women would generally want to consult their spouses or close relatives before deciding whether or not to participate in health research [50]. It is therefore very probable that female workers would want an informed consent process for occupational health purposes which enables consultations since the resulting occupational health reports could affect their employment status. In addition, the health-related challenges and needs of women are different from those of their male counterparts and this should be factored into appropriate ethical and legal frameworks for occupational health.

In light of the arguably compromised autonomy of workers in mostly poverty-stricken African settings, the difference between mandatory occupational health procedures and those which are not mandatory may be unclear. In order to protect the autonomy of vulnerable workers, it is important that guidelines and laws which clearly stipulate the modalities of occupational health surveillance are put in place in all countries. In addition, consent of workers should be sought before their personal medical information is disclosed to their employers. In order to give truly informed consent, the workers would have to read their medical reports and participate in meaningful communication that facilitates real exchange and questioning so that they fully understand the information to be disclosed, the reasons for the disclosure and possible consequences before making decisions. Compromising the privacy and confidentiality of personal medical information of workers may put their employment at stake, and if they lose their employment the repercussions are far reaching considering the extended families whose livelihoods depend on the workers. Traditional societies in Africa place heavy emphasis on the individual’s sense of personhood being based on his or her relationships with others in the community, the notion termed in Southern Nguni cultures as ‘ubuntu.’ Mechanisms to prevent paternalistic approaches and practices in occupational health, which are often the result of labour market systems that prioritise unfettered private accumulation and which encourage both management and occupational health staff to subjugate ethical considerations to production priorities, should be enhanced so as to create environments at workplaces which are conducive to the upholding of the principle of autonomy appropriate to local values. In this way, workers will be treated as ends in themselves and not as mere means to some economic end.

### Stigma and HIV-AIDS in the African work place

Stigma in occupational health practice has long been a difficult challenge. This has been particularly accentuated in dealing with HIV at the workplace, where prejudice and victimisation often lead to job loss and where stigma may accentuate profit-oriented labour practices premised on maximising efficiency. Given that Africa is the epicentre of the global HIV pandemic, and that stigma related to HIV is widespread across all sectors of society in Africa, occupational health practice in Africa is deeply affected by HIV-related stigma. Yet stigma is more than just a problem for all workplaces. It has particularly undermining implications for health in African societies.

An African saying, that a good name is better than riches, resonates with and underscores the gravity and enormity of a stigma. Stigma is associated with a bad name; it spoils a person’s social identity, and it leads to status loss. Stigma disrupts the tranquillity of a social order, disturbs the established status hierarchy, and the organized social categories of a group. This is especially so in communitarian societies, as many African societies are. The socially dissonant effect of stigma upon the African social order links stigma to the African philosophical concept of *ubuntu* or harmony, outlined above. In his exposition of an African moral theory grounded in harmony, Metz [51] observed that to be a *person* in the African ontology, or to be judged *right* in one’s action, is dependent on an agent’s friendly and positive living or relationship with others, which instantiates the philosophy of *ubuntu* or harmony in which the agent’s action identifies with others (in the sense of thinking as a ‘we’) and exhibits solidarity towards them by acting for the sake of one another.

Stigma, therefore, contradicts the binary constructs of harmony (identity and solidarity) through its potential for exclusion (failing to identify fully with the bearer as a member of a group) and discrimination (acting against, rather than promoting, the interest of the bearer). Thus, except in the unlikely event that HIV/AIDS is considered to be divisive to an African community, stigma should be considered morally wrong from an African perspective and should engage the attention of occupational health practitioners in Africa.

Situations generative of stigma include not only commercial sex work, exotic dancing, unemployment and disability, but also diseases, such as HIV/AIDS, drug-resistant TB, leprosy, urinary incontinence and mental illness [52,53]. In the African context, HIV/AIDS currently provides the primal and basal characteristic condition for stigmatization [54,55]. Etymologically, stigma was a bodily mark connoting a low social or moral status (such as slavery), or a bodily mark affixed as a religious symbol to indicate some sort of divine grace. Literally, however, stigma has come to symbolise a mark of shame or disgrace [56]. A more sociological construction and analysis of stigma emerged in the 1960s, led by the pioneering and seminal work of Goffman [57] in which he defined stigma as ‘an attribute that is deeply discrediting,’ which reduces the possessor in our minds from ‘a whole and usual person to a tainted, discounted one’. But since an attribute or a mark may be stigmatizing of one person and positively confirmatory of another, or discrediting in one context but not in another, Goffman was sceptical about the deployment of stigma as a ‘mark’ , a ‘characteristic’ or an ‘attribute’ , as if it were a thing. Thus, he suggested a relational approach, a ‘language of relationships, not attributes’, in which stigma would be construed as ‘a special kind of relationship between attribute and stereotype’. Acting on Goffman’s insights, more recent commentators have provided a definition of stigma that involves a process [55], a relationship or convergence of interrelated components [52]. Thus, Link and Phelan opined that stigma is applied when ‘elements of labelling, stereotyping, separation, status loss, and discrimination co-occur in a power situation that allows the components of stigma to unfold’ [52].

Perhaps, the element of power above, which completes the conceptualisation of the stigma process, needs emphasising. Stigma is contingent on power; stigma cannot thrive outside a relationship of power imbalance. For instance, although some of the cognitive components of stigma might be present in any stereotyping of the majority by the minority, the latter can hardly stigmatize the former as the minority lacks the power to effectuate the discriminatory effects of stigma. Consequently, Link and Phelan [50] observed that ‘stigma is entirely dependent on social, economic, and political power – it takes power to stigmatize. Similarly, Parker and Aggleton [58] observed that ‘stigma plays a key role in producing and reproducing relations of power and control’. Thus, to stigmatize is to express a sort of power-dependency relationship.

The power relations that underpin stigma comes into bold relief when consideration is given to any society that is guided by the majoritarian principle and, most especially, in African societies where the social organization is communal, and a group’s norm is given an overriding importance or priority in order to maintain the cohesion, solidarity and harmony of the group. Thus, in Africa, stigma has the potential to pierce the veil of social identity and solidarity (harmony), in that stigma is constructed as a pollutant which has contaminated the bearer, making them unacceptable, contagious and injurious to the well-being of the group and its social order; this leads to exclusion and rejection, consequences that in themselves inveigh against *ubuntu*. Interestingly, Mbonu and colleagues [54] noted the construction of HIV/AIDS in most traditional African societies as a ‘polluted disease’, a symbol of immorality, and a reflection of the victim’s sinful and evil deeds. The effects of these characterizations are certain to destabilize the tranquillity of a traditional African society or any communitarian community conscious of harmony and equality in the group. Thus, Mbonu and colleagues observed that because ‘PLWA are labelled as the “other” by the community, people try to secure the social structure, safety and solidarity by casting out offenders or reaffirming societal values’ (3). AIDS-related stigma is, therefore, morally offensive; it engenders exclusion, produces and reinforces inequality, and subjects the person living with HIV/AIDS (PLWA) to discrimination.

In the employment context, the South African case of *Hoffman v SAA*[59] is still a powerful reminder of the untoward impact of AIDS-related discrimination. In that case, an employment applicant, who had successfully gone through the four-stage selection process for employment as a cabin attendant, and who was otherwise qualified for the job, was refused employment by the South African Airways (SAA) on the ground that he tested HIV positive. Happily, the South African Constitutional Court ordered his instatement in the job, reasoning that the SAA treated the applicant unfairly and discriminatorily, without regard to the fact that the applicant’s HIV positive status would not affect his performance on the job [60]. In extreme cases, AIDS-related stigma can lead to ostracism and violence against the PLWA [61].

All this means that AIDS-related information or data in the work place (as in other places) must be kept strictly private and confidential and, unless the law permits, must not be disclosed to a third party without the permission of the PLWA. This applies as much to documented information in records as it does to the clinical interaction. Unauthorised disclosure of AIDS-related data in the work place may precipitate stigma which, in turn, produces exclusion and discrimination, outcomes that are contrary to the concept of *ubuntu* or harmony in the African philosophical thought.

### Who is the workforce?

The ICOH Ethical Code for Occupational Health Professionals states that “the aim of occupational health practice is to protect and promote workers’ health and wellbeing at work throughout their working lives…”. This is an objective well suited to countries where the majority of workers are formally employed, often unionised and are protected by various occupational health and labour regulations. However, such circumstances are the exception in Africa. Additionally, there is a very intimate relationship between the work and the home. There are many workers who live adjacent to their workplace where dangerous toxicants may pollute the home environment. Therefore, family members are also at risk of exposure to various workplace hazards. This is not unique to Africa. Indeed, the ‘father of occupational medicine’, Bernardino Ramazzini, as far back as the17th century recognised that pollution from the work environment affected people living in the vicinity of the workplace: “it appeared that many more persons died in that quarter and in the immediate neighbourhood of the laboratory than in other localities” [62]. However, in many parts of Africa, the post-colonial legacy has been that many homes are situated adjacent to the workplace.

This environmental exposure is worsened when workers’ homes are in an exposure pathway from the workplace. Several studies in Africa have demonstrated a strong association between, for example, living near asbestos mines and development of asbestosis and even mesothelioma [63,64]. In addition, some workers carry their work clothing home, thereby exposing their spouses and other family members. Hence, concentrating only at the workplace would leave out a huge number of exposed family and community members. Occupational health facilities, therefore, need to be located not only at the workplace, but also within the community where the workers reside taking full account of the social determinants of health [65].

In the African context, migrant labour is prominent in many sectors. In Southern Africa in particular, many workers from several countries flock to South Africa for work in various sectors, in particular mining and agriculture, leaving their families in their home countries. Within countries, workers also migrate from rural areas to urban areas, leaving their families behind. This type of migrant labour system with attendant overcrowded single-sex hostel accommodation exposes many workers to HIV. In the mining sector, there is the additional burden of silicosis which predisposes workers to TB. This ‘oscillating migration’ significantly increases the risk of contracting HIV and TB by family and community members far away from the workplace [66]. It is therefore imperative in the African context that the concept of who is the worker takes into consideration family and community members who may be living far away from the workplace. Such members of the extended family and community would need to be included in the provision of broad occupational health services, rather than only dealing with the individual worker who presents at the workplace.

However, Africa is not in splendid isolation. Workers come from many different backgrounds: racial, ethnic, linguistic, cultural, religious and even different countries. Many female workers are still located in the unregulated informal sector but more are entering the formal sector. The relatively paternalistic traditional African cultures and workplaces place an extra burden on the occupational health and safety of female workers. More recently there is an increasing recognition of different gender identity and sexual orientation of workers. This diversity is recognised in, for example, the South African constitution, but often, in reality at the workplace, intolerance abounds. All this poses greater challenges on the occupational health practitioner.

This paper has only been able to address some of the most cogent ethical challenges of many that emerged in debates of the workgroup. Moreover, we were unable to test some of the findings with empirical investigation, but the process has identified a number of useful research opportunities to take these ideas forward [67]. Lastly, it would have been useful to triangulate these ideas with other developing country contexts, and with non-occupational health care settings to establish a broader veracity to the finding, but this could not be accomplished in the scope of the existing project.

## Summary

Azetsop (2009) has criticised the fact that dominant paradigms in bioethics have focused on clinical care and been unresponsive to local needs in Africa, neglecting much needed population-based approaches, advocacy and health promotion to address the social determinants of health [68]. We concur inasmuch as we believe there is a need to move Ethical Guidelines into addressing questions of power and vulnerability, both local, as they play out in relationships between employers and workers, and amongst workers, and global, in that occupational health practitioners should recognise global injustice and inequity as an ethical matter. Not insignificantly did the Chair of Commission on Social Determinants of Health comment that “The unequal distribution of health-damaging experiences is not in any sense a ‘natural’ phenomenon but is a result of a toxic combination of poor social policies and programmes, unfair economic arrangements, and bad politics” [65]. Our experience in Africa points to the need for Ethical Codes to challenge inequalities in power at the workplace, rather than assuming them as given.

Secondly, the principle arising from African traditional philosophy that “authentic personhood require[s] being in relationship with others” [49] provides a powerful lever to rethinking bioethical approaches to consent, stigma and the scope of occupational health practice. Operationalising the idea that ‘a person is a person through other persons’ in our Ethical Codes will harmonize and balance individual and community needs and lend a stronger moral authority to the kinds of ethical guidelines adopted in developing countries, as well as being more likely to “protect and promote workers’ health and wellbeing at work throughout their working lives” in an African context.

Lastly, citizens are able to claim their rights from states, not from international systems. Basic human rights may be considered as a set of values within universal reach. They assume, however, real importance for individuals and groups to the extent that they are actualised in states respecting human rights and assuming responsibility for their obligation, as well as to the extent that rights-holders are active agents in claiming their rights. This unique yet dynamic African environment demands solutions from within Africa and highlights the responsibility of governments in Africa, as much as in other developing countries, to adapt international norms to the specific situation of their country or to draft complementary regulations to supplement international guidelines. In response to the Universal Declaration of Human Rights, the continent created the African Charter on Human and Peoples Rights which came into effect in 1986. Perhaps an analogous development might be the need to evolve an Afro-centric African Charter on Bioethics that is able to draw on both ‘Western’ and traditional African philosophy in its mission to protect and promote occupational health globally.

## Competing interest

The authors declare they have no financial conflict of interest in the work published here. Three of the authors (LL, PW and RMH) are members of ICOH and PW is an ICOH office bearer.

## Authors’ contribution

RN contributed to the discussion to generate the manuscript, participated in drafting sections of the manuscript and commented on revisions. AN contributed to the discussion to generate the manuscript, participated in drafting sections of the manuscript and commented on revisions. PW helped to establish the Working Group, contributed to the discussion to generate the manuscript, participated in drafting sections of the manuscript and commented on revisions. RM contributed to the discussion to generate the manuscript, participated in drafting sections of the manuscript and commented on revisions. NM contributed to the discussion to generate the manuscript, participated in drafting sections of the manuscript and commented on revisions. GT contributed to the discussion to generate the manuscript, participated in drafting sections of the manuscript and commented on revisions. LL conceived of the study, convened the working group, coordinated and participated in the discussion to generate the manuscript and led the drafting and revisions of the manuscript. All authors read and approved the final manuscript. While this workgroup was assembled under the International Commission on Occupational Health (ICOH), the views expressed in this manuscript are entirely those of the authors and do not reflect the views of ICOH or any of its structures.

## Pre-publication history

The pre-publication history for this paper can be accessed here:

http://www.biomedcentral.com/1472-6939/15/48/prepub
